# Flower morphology, flower color, flowering and floral fragrance in *Paeonia* L.

**DOI:** 10.3389/fpls.2024.1467596

**Published:** 2024-11-21

**Authors:** Yongming Fan, Xing Jin, Mengshan Wang, Huadong Liu, Weili Tian, Yandong Xue, Kai Wang, Hu Li, Yan Wu

**Affiliations:** ^1^ College of Architecture, North China University of Water Resources and Electric Power, Zhengzhou, China; ^2^ Construction Decoration Co., LTD of China Construction No.7 Engineering Bureau, Zhengzhou, China

**Keywords:** *Paeonia*, floral organ, flower development, regulatory mechanism, research progress

## Abstract

*Paeonia* have diverse flower colors, rich flower types, varying bloom periods, and delightful fragrances, rendering them highly valuable for both ornamental and economic purposes in horticulture. Investigating the developmental mechanisms of morphology, flower color, flowering and floral fragrance in *Paeonia* holds significant value for enhancing their ornamental traits and conducting germplasm improvement. This review provides an overview of research progress on *Paeonia* flower morphology (including flower bud differentiation, classification, omics applications in shape studies, and functional genes regulating flower morphology), flower colors (omics applications in color research and functional genes regulating flower colors), bloom periods (flower bud dormancy, flowering time), and fragrances (preparation, analysis, components, and molecular biology research of flower fragrances) within the *Paeonia*. Additionally, it offers a comprehensive analysis of current research challenges and future directions.

## Introduction

**Figure 1:**
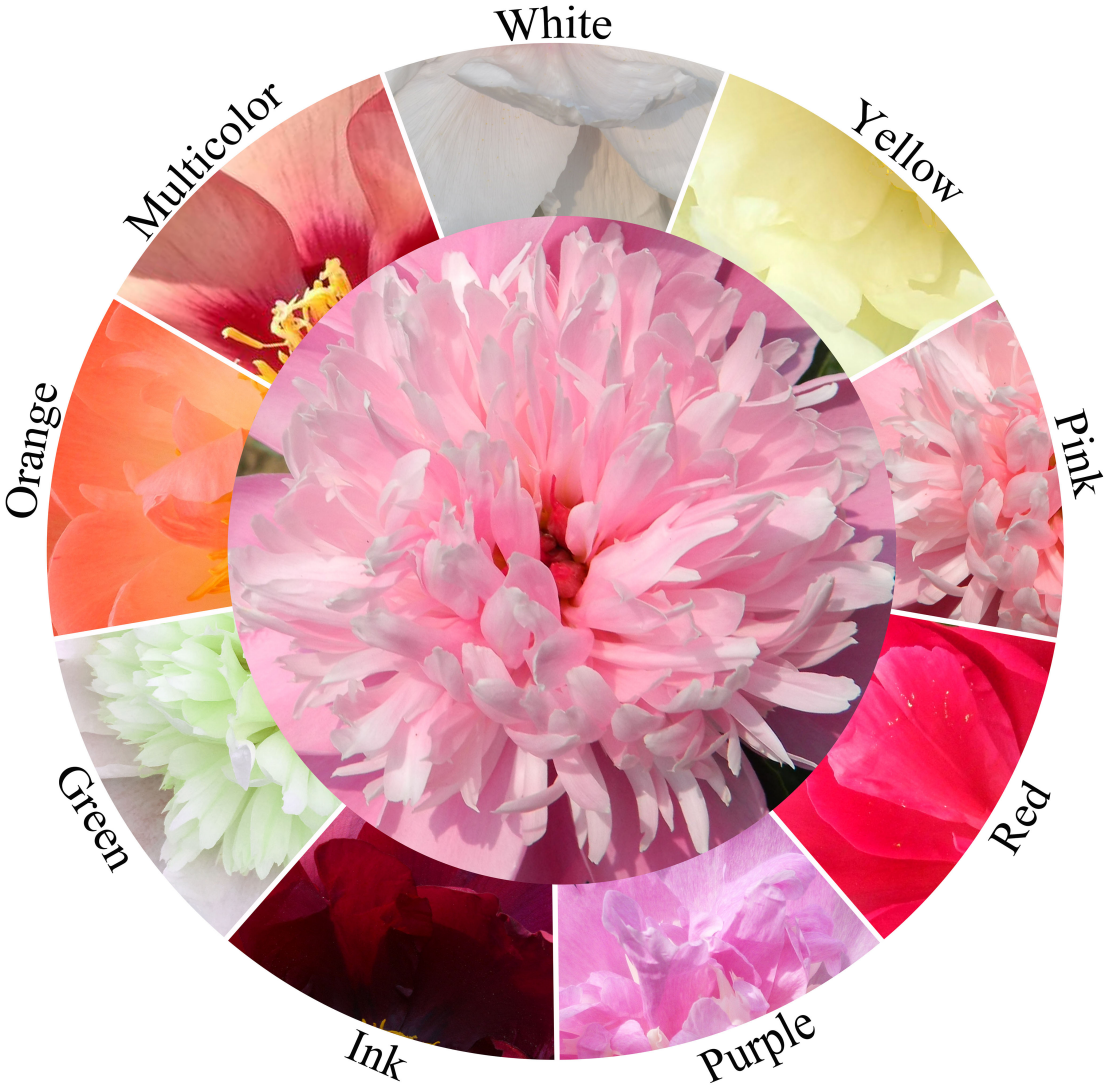
Nine different colors of herbaceous peony flowers.

*Paeonia* L. is the only genus in Paeoniaceae and it holds great ornamental and economic value in gardening. The flower is one of the key characteristics that contribute to the ornamental and economic value of *Paeonia*. Factors that influence the aesthetic and economic value of the flower include its morphology, color, flowering, and floral fragrance. *Paeonia* exhibit a diverse range of flower morphologies, with a total of 13 different forms (Fan et al., 2020, 2023a). This variety of flower morphologies provides people with a diverse visual experience, enhancing its appeal as an ornamental plant. The flower color of *Paeonia* plays an important role in determining its ornamental value. Herbaceous peony flowers are known to have nine major color series, including white, yellow, pink, red, purple, ink, green, orange, and multicolor (**Figure 1**). The flowering period of *Paeonia* can be divided into five categories: extremely early flowering, early flowering, mid-flowering, late flowering, and extremely late flowering. *Paeonia* vary greatly in their flowering time and duration. By strategically combining different cultivars in garden design, can create a *Paeonia* garden with an extended blooming season. Additionally, the fragrant flowers not only provide a delightful sensory experience but also have economic value for use in floral arrangements, sachets, and other decorative purposes.

Differences in flower morphology of *Paeonia* begin with flower bud differentiation. Methods commonly used to study the differences between flower types include transcriptomics and proteomics. These studies have identified transcription factors and functional genes that may be involved in regulating flower development, providing important insights into the molecular mechanisms underlying flower development in *Paeonia* (Fan et al., 2020). In the study of flower color, molecular biology approaches have been widely used to explore the regulation of anthocyanins, flavonoids, flavonols, and other flavonoid substances in Paeonia flowers, identifying the functions of related genes (Guo et al., 2019; Zhang et al., 2018a). In research on flowering time, genes regulating flowering, including bud dormancy, bud differentiation, and flower senescence, have been identified (Wu et al., 2018). Studies on floral fragrance have focused on identifying the aromatic components of *Paeonia* and uncovering genes that may be involved in regulating scent synthesis (Zhao et al., 2023). Although numerous scholars have conducted research on flower morphology, color, flowering time, and floral fragrance in *Paeonia*, the relationships between these studies are not yet clear. The interactions among identified genes remain unclear, and the research results on the floral organs of *Paeonia* are not yet systematic.

This review systematically summarizes important research related to floral traits in *Paeonia*, including flower morphology (flower bud differentiation, flower type classification, application of genomics in flower type research, and functional genes regulating flower morphology), flower color (application of genomics in flower color research, and functional genes regulating flower color), flowering time (flower bud dormancy, and flowering time), and floral fragrance (preparation, analysis, and components of floral fragrance, and molecular biology research on floral fragrance). The compilation in this review can serve as a reference for researchers in the field of *Paeonia* to conduct studies related to floral trait improvement.

## Flower morphology

### Floral type classification

In China, the classification of flower types within the *Paeonia* exhibits some differences compared to other countries. According to Qin and Li (1990), the flower types of *Paeonia* were divided into three levels according to the series, class, and type. According to the differences between the wild species of tree peony, cultivars were divided into peony series, spotted peony series, yellow peony series and purple peony series. According to the differences between wild species or populations of herbaceous peony, cultivars were divided into Chinese peony series and European peony series.

Based on the differences in flower structure, tree peonies and herbaceous peonies were further classified into two major categories within each group: single-flower type and double-flower type. Within the single-flower type, they were further divided into the Thousand-layer Subclass and the Lou-zi Subclass based on the origin of the petals, totaling nine flower types. These included Single form, Lotus form, Chrysanthemum form, Rose form, which belong to the Thousand-layer Subclass, and Golden-slamen form, Anemone form, Golden-circle form, Crown form, Globular form, which belong to the Lou-zi Subclass. The double-flower types were divided into the Thousand-layer Tai-ge Subclass and the Lou-zi Tai-ge Subclass, totaling four flower types, including Primary Proliferation form, Color-petalled Proliferation form, Stratified Proliferation form, and Globular Proliferation form. Primary Proliferation form belonged to the Thousand-layer Tai-ge Subclass, while Color-petalled Proliferation form, Stratified Proliferation form, and Globular Proliferation form belonged to the Lou-zi Tai-ge Subclass (Qin and Li, 1990).

**Figure 2:**
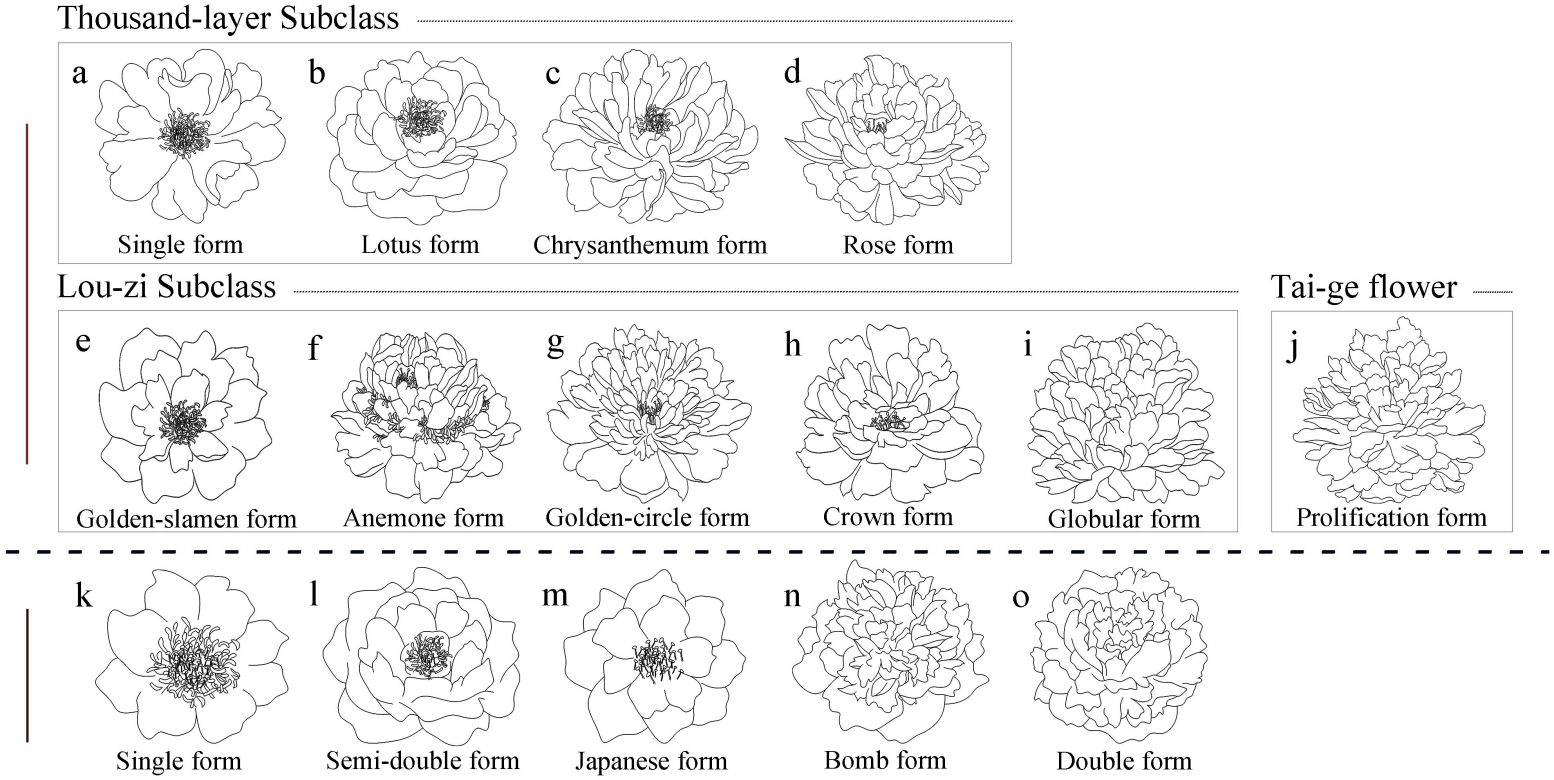
Flower type classification of *Paeonia* in China and America. **(A-J)** represent the flower type classification of *Paeonia* in China; **(K-O)** represent the flower type classification of *Paeonia* in America; a-d are the Thousand-layer Subclass; **(E-I)** are the Lou-zi Subclass, **(J)** is the Tai-ge flower.

Different flower types exhibit distinct morphological differences. Single-petal type: 2-3 rounds of petals, normal development of stamens and pistils (**Figure 2A**). Lotus type: 4-5 rounds of petals, normal development of stamens and pistils (**Figure 2B**). Chrysanthemum type: Over 6 rounds of petals, reduced number of stamens, some stamens petaloid (**Figure 2C**). Rose type: Extreme proliferation of petals, complete loss of stamens or several small stamens in the flower center (**Figure 2D**). Golden stamen type: Outer petals expanded, 2-3 rounds of petals, stamens dome-shaped (**Figure 2E**). Tulip type: Outer petals 2-3 rounds, stamens completely petaloid, pistils normal (**Figure 2F**). Golden ring type: Wide outer petals, stamens transformed into elongated inner petals, a circle of normal stamens remaining between inner and outer petals (**Figure 2G**). Crown type: Wide outer petals, stamens almost completely petaloid, rounded and prominent (**Figure 2H**). Hydrangea type: Stamens completely petaloid, forming petals, resembling a hydrangea (**Figure 2I**).

Tai-ge flowers consist of two flowers: an upper flower and a lower flower (**Figure 2J**). In the initial Tai-ge type, both upper and lower flowers develop normal stamens and pistils, with little difference in petal morphology between inner and outer petals. In the variegated petal Tai-ge type, a few stamens are petalized, some stamens and pistils develop normally in the upper flower, while the pistils of the lower flower are almost completely petalized, with some differences in petal shapes between inner and outer petals. In the layered Tai-ge type, stamens in the upper flower are almost completely petalized or few remain, while pistils develop normally; in the lower flower, both stamens and pistils are almost completely petalized, with noticeable differences in petal shapes between inner and outer petals. In the spherical Tai-ge type, stamens and pistils in both upper and lower flowers are almost completely petalized, with differences in petal shapes between inner and outer petals. The classification of flower types is based on their growth and development stages; once the flowers are fully open, it becomes more difficult to accurately identify the type of Tai-ge flower.

The American Peony Society classifies the flower forms of the *Paeonia* into five types based on floral structure, including Single form (**Figure 2K**), Semi-double form (**Figure 2L**), Japanese form (**Figure 2M**), Bomb form (**Figure 2N**), and Double form (**Figure 2O**).

### Flower bud differentiation

Flower bud differentiation refers to the process in which the growth status of a plant changes from vegetative bud to reproductive bud. The apical meristem of the bud undergoes a transition from vegetative growth state to reproductive growth state under suitable conditions. When the plant receives a signal to initiate flowering, the apical meristem of the bud transforms into an inflorescence meristem, and then the floral organs begin to develop (Coen and Meyerowitz, 1991). During the nutritional growth process of *Paeonia*, the apex of the stem (or bud) is pointed in the vegetative growth stage. Upon entering the reproductive growth stage, the apex of the stem (or bud) becomes relatively blunt. The protrusion formed at the edge of the apical meristem is the bract primordium. After the formation of the bract primordium, protuberances gradually appear on its inner side, which are the sepals primordium. As the floral bud continues to develop, the primordia of petals gradually appear on the inner side of the sepals primordium, and the layers of petal primordia correspond to the number of petals. The stamen primordium begins to appear after the formation of petal primordia. Before the stamen primordium is fully formed, the carpel primordium also begins to develop. The process can be divided into six stages: floral bud differentiation initiation stage, bract primordium differentiation stage, sepal primordium differentiation stage, petal primordium differentiation stage, stamen primordium differentiation stage, and carpel primordium differentiation stage (**Table 1**) (Zhang et al., 2019). Herbaceous peony and tree peony have the same floral bud differentiation process, but the tree peony floral bud differentiation period is earlier and the process takes less time compared to herbaceous peony (He et al., 2014). In terms of research methods, the main approaches used to observe floral bud differentiation include paraffin sectioning, cryosectioning, and stereomicroscopy (Zhang et al., 2019). Some studies suggest that paraffin sectioning provides clearer images than cryosectioning, while stereomicroscopy is the simplest and quickest among the three methods (Zhang et al., 2019). Research on floral bud differentiation not only allows for an understanding of the process in *Paeonia* from a morphological perspective but also summarizes the regularities in floral pattern formation. It provides reference for sampling in further molecular biology studies (Fan et al., 2021). Additionally, studying floral bud differentiation helps in understanding the floral types of *Paeonia*, providing guidance for selecting parents in hybrid breeding programs (He et al., 2014).

**Table 1:** Floral bud differentiation processes in herbaceous peony and tree peony.

Cultivars	Flower type	Flower bud differentiation process	Observation method	References
*P. lactiflora* ‘Sarah Bernhardt’	Prolofication form	Bracts primordium differentiation period, sepals primordium differentiation period, petals primordium differentiation period	Scanning electron microscope	Barzilay et al., 2002
*Paeonia lactiflora* ‘Fen Yu Nu’	Simple form	Early flower bud differentiation, bracts primordium differentiation period, sepals primordium differentiation period, petals primordium differentiation period, stamens primordium differentiation period, and pistillodes primordium differentiation period	Paraffin sections	He et al., 2014
*P. suffruticosa* ‘Feng Dan Bai’	Crown form			
*P. lactiflora* ‘Da Fu Gui’	Prolofication form		Paraffin sections and microscopic observations by stereomicroscopy	Zhang et al., 2019
*P. suffruticosa* ‘Yu Mian Tao Hua’	Chrysanthemum form		Paraffin sections	Dong et al., 2020
*P. suffruticosa* ‘Liu Li Guan Zhu’	Globular form			
*P. suffruticosa* ‘Hong Li Li’	Rose form			
*P. rockii* ‘Guan Gong Hong’	Single-petal flower and Double-petal flower		Paraffin sections	Zhang et al., 2022a

#### Application of genomics in floral morphology research

With the advancement of molecular biology techniques, research on flower organ development in *Paeonia* is increasing (Fan et al., 2020). The genome of *Paeonia suffruticosa* ‘Luo Shen Xiao Chun’ has been sequenced and published. Genome assembly and *de novo* transcriptome assembly identified a total of 77 MADS-box genes, including 44 type I and 33 type II genes. Among the type II genes, 18 were identified as ABCDE genes, comprising 5 A class genes, 5 B class genes, 2 C/D class genes, and 6 E class genes. Among the 5 B class genes, apart from the *PISTILLATA*, B*-PI* (*PsuMADS6*) gene, the other four B class genes showed high expression levels in flowers. The *APETALA3* (*AP3*) gene (*PsuMADS32* or *TRINITYpsu14*) and B*-PI* (*TRINITYpsu13*) exhibited similar expression levels in flowers and flower buds, suggesting that their encoded proteins form heterodimers during petal and stamen development. The *TOMATO MADS-BOX 6*, B-*TM6* played a crucial role in stamen petaloidy and floral morphogenesis, indicating its partial functionality in C-class genes. Studies have shown that C-class gene expression levels are low in floral organs of *P. suffruticosa* ‘Luo shen xiao chun’, suggesting a potential association with observed stamen petaloidy in this peony cultivar. When C-class gene functions were limited, *TM6* expression ensures proper carpel development. The research speculated that *AP3/PI* and *TM6* genes together determined tree peony petal and stamen formation, as well as their functional interconversion when C-class gene function was restricted (Lv et al., 2020). Additionally, the genome of *P. ostii* has been sequenced, with a genome size exceeding 10 Gb. Studies indicated that ectopic expression of A-class gene *APETALA1* (*AP1*) and reduced expression of C-class gene *AGAMOUS* (*AG*) might have contributed to petaloid stamen formation (Yuan et al., 2022).

Transcriptome, proteome, microRNA, and degradome studies have been extensively applied in the research of floral organ development (**Table 2**). In tree peonies, omics studies have identified several genes belonging to the MADS-box gene family, including *PsAP1*, *APETALA2* (*PsAP2*), *PsAP3*, *PsPI*, *PsAG*, *SEPALLATA3* (*PsSEP3*), and *AGAMOUS-LIKE* (*AGLs*), categorized as A, B, C, and E class genes within the ABCDE model of floral organ development (Wang et al., 2019). For instance, a transcriptome sequencing study of petals from *P. suffruticosa* ‘Luo yang hong’ at bud and full blooming stages identified 71 candidate genes across 4 groups potentially linked to tree peony petal formation or development. Specifically, Group 1 (*MADS-box* genes, *SQUAMOSA promoter-binding-protein* (*SBP*) genes, *teosinte branched1/cycloidea/proliferating cell factors* (*TCP*) genes) and Group 2 (*trihelix* genes, *basic region/leucine zipper motif* (*bZIP*) genes, *B_3_* genes, *cysteine-rich polycomb-like protein* (*CPP*) genes) directly influence floral organ formation, while Group 3 (*light signal transduction-related* genes) and Group 4 (*plant hormone-related* genes) were involved in light signal transduction-mediated and plant hormone-mediated processes during floral organ development (Li et al., 2017). Furthermore, members of transcription factor families such as *basic helix-loop-helix* (*bHLH*), *GRAS*, *homeodomain-leucine zipper* (*HD-ZIP*), *leucine-rich repeat* (*LRR*), v-myb avian myeloblastosis viral oncogene homolog (*MYB*), *NAC*, Ring Finger Protein 1 (*RING1*), *Trihelix*, *WD*, and *WRKY* also played crucial roles in peony floral development (Liu et al., 2019; Guo et al., 2022).

**Table 2:** The application of omics in the study of floral organ development.

Genomics	Research materials	Research overview	References
Transcriptomic	Petals at bud stage and full blooming stage from *P. suffruticosa* ‘Luo Yang Hong’	71 genes related to petal morphogenesis were identified, including four groups (Group 1: *MADS-box*, *SBP*, *TCP*; Group 2: *trihelix*, *bZIP*, *B_3_*, *CPP*; Group 3: light signal transduction-related genes; Groups 4: plant hormone-related genes).	Li et al., 2017
Transcriptomic	Flower buds from *P. suffruticosa* ‘Hu Chuan Han’, ‘High Noon’, ‘Luoyang Hong’, ‘Zi Luo Lan’, and *P. delevayi*	Genes involved in the development of floral organs were identified. Sepal development (*PsAP1* and *PsAP2*), stamen and pistil development (*PsAG*), *PsSEP3* and *PsSEP4* involved in the development of four-wheel floral organs, and other *AGLs* genes.	Wang et al., 2019
Transcriptomic	Flower buds from *P. rockii* cultivars (JS and FM)	6 floral organ development genes (*PsAGL11*, *PsPI2*, *PsAP2-like ANT*, *PsAP1*, *PsAIL1*, *PsAGL9*), 6 transcription factors (*PsMYB114-like*, *PsMYB12*, *PsMYB61-like*, *PsNAC*, *PsWRKY13*, *PsWD76-like/2*) and 3 enzyme-like genes (*PsRING1*, *PsRING1a-3*, *PsLRR GSO2*) were identified that may be involved in quantitative variation of tree peony carpel.	Liu et al., 2019
miRNAome, transcriptomic and degradome	Fresh petals from *P. ostii* ‘Feng Dan’, mutant line of *P. ostii* ‘Feng Dan’, and *P. suffruticosa* ‘Lian He’	*MYB*-related, *bHLH*, *trihelix*, *NAC*, *GRAS* and *HD-ZIP* transcription factor families were involved in the process of floral florescence, development, and senescence of tree peony.	Guo et al., 2022
Transcriptomic and proteomic	Outer and inner petals from *P. lactiflora* ‘Zi Feng Yu’	*PlAP2* played an important role in the formation of herbaceous peony petals.	Wu et al., 2018
Transcriptomic	Flower buds from *P. lactiflora* ‘Fen Yu Nu’ and ‘Lian Tai’	Seven MADS-box genes, *PlAP3*, *PlDEFA*, *PlPI2*, *PlAG-1*, *PlSEP3*, *PlSEP1-1*, and *PlSEP1-2*, and 11 other transcription factors, *PlTCP2*, *PlTCP4*, *PlTCP9*, *PlbHLH36*, *PlICE1*, *PlLBD38*, *PlNAC083*, *PlBLH11*, *PlPDF2*, *PlGBF1*, and *PlIIIA* were likely involved in the formation and development of petaloid stamens.	Fan et al., 2021
Transcriptomic and proteomic	Flower buds from *P. lactiflora* ‘Fen Yu Nu’ and ‘Lian Tai’	The metabolic pathways related to polysaccharides, carbohydrates, starch, sucrose, fructose, mannose, and photosynthesis may be involved in petaloid stamens.	Fan et al., 2023a

In herbaceous peonies, floral organ development genes identified through genomic studies were predominantly members of the MADS-box gene family, including *PlAP3*, *PlDEFA*, *PlPI2*, *PlAG-1*, *PlSEP3*, *PlSEP1-1*, and *PlSEP1-2*. Additionally, nine other transcription factor families were also participate in herbaceous peony floral organ development, namely *TCP*, *bHLH*, *ICE*, *LBD*, *NAC*, *BLH*, *PDF*, *GBF*, and *IIIA* (Wu et al., 2018; Fan et al., 2021). Genomic research has identified candidate genes involved in *Paeonia* floral development, laying a foundation for further studies on gene function.

#### Functional genes regulating floral morphology

**Figure 3:**
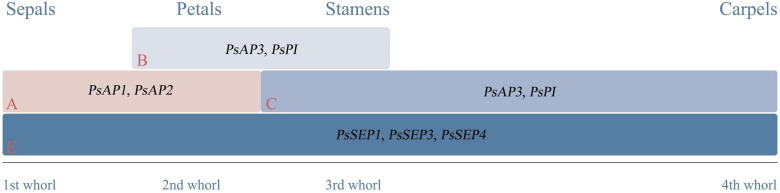
ABCE model of tree peony flower organ development.

In studies related to functional genes involved in floral organ formation in tree peony, genes expressed in sepals included *PsAP1*, *PsAGL6*, and *PsMADS5*. Genes expressed in petals included *PsAP1/PsAP1fd*, *PsAP2*/*PsAP2fd*, *PsAP3*/*PsAP3fd*, *PsPI/PsPIfd*, *PsMADS1*, *PsMADS5*, *PSMADS9*, and *PsAGL6*. Genes expressed in stamens included *PsAG/PsAGfd*, *PsAP3/PsAP3fd*, *PsMADS1*, *PsPI/PsPIfd*, *PsMADS5*, *PsAGL6*, and *PsTM6*. Genes expressed in carpels/ovules included *PsAP1*, *PsMADS1*, *PsAG*/*PsAGfd*, *PsMADS5*, and *PsAGL6*. Additionally, E-class genes *PsSEP1/PsSEP1fd*, *PsSEP3*, and *PsSEP4* were expressed in four floral organs. These genes collectively participate in the process of organ formation in peony flowers (Ren, 2011; Shu et al., 2012; Tang et al., 2018a; Zhang et al., 2015a; Wang et al., 2019; Zhou et al., 2022). Research indicated that in tree peonies, *PsAP1* and *PsAP2* were primarily expressed in sepals and bracts, with weak expression in petals and carpels, and almost no expression in stamens. *PsAP3* and *PsPI* exhibit strong expression in petals and stamens, with lower expression in sepals and carpels. *PsAG* showed high expression in stamens and carpels. *PsSEP1*, *PsSEP3*, and *PsSEP4* were expressed in all four floral organs. This suggested that *PsAP1* and *PsAP2* were involved in sepal development, *PsAP3* and *PsPI* in petal and stamen development, and *AG* in stamen and carpel development. *PsSEP1*, *PsSEP3*, and *PsSEP4* participated in the development of all four floral organs (Wang et al., 2019). In *P. ostii* ‘Feng Dan’, the A-class gene *PsAP1fd* was mainly expressed in sepals and petals, while *PsAP2fd* was predominantly expressed in tree peony petals, indicating their likely involvement in sepal development. The B-class genes *PsAP3fd* and *PsPIfd* exhibited strong expression in stamens and petals, respectively, with *PsPIfd* showing the highest expression in petals, suggesting their roles in petal and stamen development. The C-class gene *PsAGfd* showed the highest expression in carpels. The E-class gene *PsSEP1fd* was predominantly expressed in carpels (Zhou et al., 2022). The expression patterns of these genes in tree peonies demonstrate that the regulation of floral organ development follows a predictable pattern according to the ABCE model (**Figure 3**).

In herbaceous peonies, the expression levels of A-class gene *PlAP1* was highest in sepals, lower in petals, and very low in stamens and carpels. The expression of *PlAP2* gene was highest in carpels, lower in sepals, and very low in petals and stamens. Among B-class genes, *PlAP3-2* and *PlPI* showed highest expression in stamens, lower in petals, and very low in sepals and carpels. *PlAP3-1* exhibited descending expression levels across floral organs, highest in petals, followed by stamens, carpels, and sepals, with its expression in floral organs 100 times lower than that of *PlAP3-2*. *PlSEP3* was expressed in all floral organs, with levels highest in sepals, followed by carpels, petals, and stamens (Ge et al., 2014). Additionally, research indicated that AP3-PI obligate heterodimerization played a crucial role in the determination of organ identity in corolla and stamens (Gong et al., 2017).

With deeper research, certain genes related to floral organ development in *Paeonia* have been isolated, while studies on functional genes governing tree peony flower development are more advanced compared to those on herbaceous peony. Although the functions of some genes have been verified to participate in the process of peony floral organ development (**Table 3**), their specific roles across different floral organs and their interactions with other genes remain unclear.

**Table 3:** Functions of ABCE class genes in *Paeonia.*.

Class	Function	References
A	Involved in the development process of sepals and petals	Ren, 2011; Shu et al., 2012; Tang et al., 2018a; Zhang et al., 2015a; Wang et al., 2019; Zhou et al., 2022
B	Involved in the development process of petals and stamens	
C	Involved in the development process of stamens and carpels	
E	Involved in the entire process of forming the four whorls of floral organs	

## Flower color

### Application of omics in the study of floral color

In *Paeonia*, the different flower colors are closely associated with the types of pigments present in petal cells, cellular pH values, epidermal cell shapes, and intracellular physiological environments. The main determinants of flower color are the types and concentrations of pigments. *Paeonia* flowers primarily contain anthocyanins, flavonoids, flavonols, and other flavonoid substances, among which anthocyanins play a key role in determining flower color (Wang et al., 2023). With the advancement of molecular biology technologies, applications of omics such as transcriptomics, proteomics, microRNA analysis, Simplified Genome-Specific Locus Fragment Sequencing (SLAF-seq), DNA methylation profiling, and others have provided effective research methods for elucidating the biosynthesis pathways and regulatory genes involved in flavonoid and anthocyanin glycoside production related to *Paeonia* flower colors. These approaches also facilitate the discovery of new genetic resources and the clarification of genetic evolution patterns.

In studies of flower color in *Paeonia*, transcriptomics was the most widely utilized approach, while proteomics, microRNA, DNA methylation, and SLAF-seq were less commonly applied. Research indicated that multiple genes were involved in tree peony flower color formation, including *flavanone 3-hydroxylase* gene (*F*_3_ *H*), *dihydroflavonol 4-reductase* (*DFR*), *anthocyanidin synthase* (*ANS*), *anthocyanidin 3-O-glucosyltransferase* (*3GT*), *THC 2′-glucosyltransferase* (*THC2’GT*), *SQUAMOSA promoter-binding protein-like* (*SPL2*), *flavone synthase*, (*FNS II*), *chalcone synthase* (*CHS*), *chalcone isomerase* (*CHI*), *F_2_H*, flavanone-3-hydroxylase (*F_3_′H*) and *PsDFR*, among others. These genes regulated the synthesis of flavonoids and anthocyanins, thereby influencing flower color presentation (Shi et al., 2015; Luo et al., 2022; Zou et al., 2023; Zhang et al., 2018a). In addition, some transcription factors *MYB*, *bHLH* and *WD40* were also involved in the regulation of floral color (Gu et al., 2019. *PoMYB2* and *PoSPL1* were reported to negatively regulate the accumulation of anthocyanins by modulating the activity of the MYB-bHLH-WDR complex (Gao et al., 2016). Among different-colored peonies, numerous genes showed differential expression, potentially directly or indirectly participated in the formation and regulation of floral color. *UDP-glucosyltransferases* (*UGTs*) such as *PhUGT78A22* was reported to play important roles in floral color formation, participating in glycosylation reactions of flavonoids and anthocyanins (Li et al., 2022a). Methylation modifications played crucial regulatory roles in the formation of spots on tree peony petals, potentially influencing the expression of structural genes (Zhu et al., 2023) (**Table 4**). Key genes and regulatory mechanisms influencing the formation of flower color in herbaceous peonies primarily focus on the biosynthetic pathways of flavonoids and anthocyanins, as well as the regulatory roles of transcription factors. Genes such as *phenylalanine ammonialyase* gene (*PlPAL*), *flavonol synthase* gene (*PlFLS*), *PlDFR*, *PlANS*, *Pl3GT*, and *anthocyanidin 5-O-glucosyltransferase* gene (*Pl5GT*) were involved in the biosynthetic pathways of flavonoids and anthocyanins, playing crucial roles in the formation of yellow flower color (Zhao et al., 2014). Transcription factors *PsMYB111*, *PsMYB4* and *PsSPL9* regulated the expression of structural genes, influencing the synthesis and accumulation of pigments in herbaceous peony flowers. Research had found that by modulating the expression levels of specific genes, it was possible to induce or inhibit the accumulation of particular pigments, thereby affecting the presentation of herbaceous peony flower colors (Luo et al., 2021a). Transcriptome sequencing and association analysis have revealed SNPs closely associated with herbaceous peony flower color, providing crucial information for genetic improvement of herbaceous peony coloration (Liu et al., 2022). *miR156e-3p* might participate in the regulation of yellow flower color formation by modulating specific genes such as *SPL1* (Zhao et al., 2017).

**Table 4:** The application of omics in the study of flower color development.

Genomics	Research materials	Research overview	References
Transcriptomic	Purple-red-flowered and yellow-flowered from *P. delavayi*	Up-regulated of *F_3_H*, *DFR*, *ANS* and *3GT* genes might have played an important role in purple-red petal pigmentation, while high co-expression of *THC2’GT*, *CHI* and *FNS II* were key factors for carotenoid accumulation in yellow petals.	Shi et al., 2015
Transcriptomic	Petals were divided into 4 categories according to the color intensity level, and 4 petals were taken from each color intensity level in *P. ostii*	The expression of anthocyanin biosynthetic genes was positively correlated with anthocyanin concentration in flowers, while *PoMYB2* and *PoSPL1* might have negatively regulated anthocyanin accumulation by influencing the activation ability of MYB-bHLH-WDR complex.	Gao et al., 2016
Transcriptomic	Red and white petals from *P. suffruticosa* ‘Shima Nishiki’	The significant differential expression of *PsDFR*, *PsMYB* and *PsWD40* likely played an important role in anthocyanin concentration in red and white petals, thereby mediating bicolor formation.	Zhang et al., 2018a
Transcriptomic	Four developmental stages flowers from *P. suffruticosa* ‘Qing Hai Hu Yin Bo’	*PsMYB12* interacted with *PsbHLH* and *PsWD40* to form a protein complex, which directly activated the expression of *PsCHS*, which was unique in the formation of petal spots.	Gu et al., 2019
Transcriptomic	Four flowing stages yellow flowers from *P. delavayi* var. lutea	The overexpression of *PdCHS*, *PdTHC2’GT* and the inhibition of *PdCHI* expression increased the accumulation of *PdISP*. The overexpression of *PdLCYB*, *PdCHYB* and the inhibition of *PdCCD* expression enhanced lutein accumulation. *PdCHS* and *PdCHI* were regulated by *PdMYB2*.	Zou et al., 2023
microRNA	Five blooming stages petals from *P. suffruticosa* ‘High Noon’ (yellow flowers) and ‘Rou Fu Rong’ (purple-red flowers)	The overexpression of *PsSPL2* from tree peony in tobacco caused a decrease in anthocyanin content, with downregulation of *NtF_3_′H* and *NtDFR*. Silencing *PsSPL2* in tobacco resulted in pale yellow petals. Additionally, silencing of *PsSPL3* significantly decreased the expression levels of *PsCHS*, *PsCHI*, and *PsF_2_H* in petals, while significantly increasing the expression levels of *PsF3′H* and *PsDFR.*	Luo et al., 2022
Transcriptomic	Blotched and non-blotched parts flowers from *P.* ‘He Xie’ (intersectional hybrid between the section Moutan and *Paeonia*)	A key UDP-glycosyltransferase named *PhUGT78A22* was identified, silencing of *PhUGT78A22* reduced the content of anthocyanidin 3,5-O-diglucoside in *P.* ‘He Xie’.	Li et al., 2022a
Transcriptomic and DNA methylation	Purple and white areas of petals were analyzed from *P. rockii* ‘Shu Sheng Peng Mo’	The flavonoid metabolic pathway enzyme genes *PrF_3_H*, *PrDFR* and *PrANS* were key enzyme genes determining the differential pigment synthesis inside and outside the spots. The high abundance of methylation modifications was likely an important regulatory mechanism mediating the silencing of the enzyme genes *PrF_3_H* and *PrANS* outside the spots.	Zhu et al., 2023
Transcriptomic	Red outer-petals and yellow inner-petals from *P. lactiflora* ‘Jin Hui’	*PlPAL*, *PlFLS*, *PlDFR*, *PlANS*, *Pl3GT* and *Pl5GT* were likely involved in the flavonoid biosynthetic pathway that contributed to the formation of yellow flowers and participated in the yellow formation process mediated by peonidin biosynthesis.	Zhao et al., 2014
microRNA	Red outer-petals and yellow inner-petals from *P. lactiflora* ‘Jin Hui’	The yellow formation might have been regulated by miR156e-3p-targeted squamosa promoter binding protein-like gene (*SPL1*).	Zhao et al., 2017
Transcriptomic	Seven flowering stages petals from *P. lactiflora* ‘Coral Sunset’ and ‘Pink Hawaiian Coral’	Eight structural genes *CHS*, *F_3_H*, Flavonoid 3′-hydroxylase gene (*F_3_’H*), *FLS*, *ANS*, *ANR*, and UDP glucose: flavonoid-3-O-glucosyltransferase (*UFGT*) and 13 transcription factors (five *MYB*, three *bHLH*, one *WD40*, one *HY5*, one *PIF3*, one *COP1* and two *PHY*) that were associated with flavonoid biosynthesis and participated in the process of peony flower color change.	Guo et al., 2019
Full-length transcriptomics and metabolomics	Petals of pure yellow flowers *P. suffruticosa* ‘High Noon’ and pure-red flowers ‘Roufurong’ in five flowering stages	*PsMYB111* influenced the accumulation of flavonols by directly regulating the expression of *PsFLS* and reducing the flux of anthocyanin synthesis. *PsMYB4* might have interacted with *PsEGL3* to negatively regulate certain structural genes, leading to a reduction in anthocyanin synthesis. *PsSPL9* might have negatively regulated *PsDFR* independently to inhibit the accumulation of anthocyanins.	Luo et al., 2021a
SLAF-seq	DNA was extracted from the leaves and petals were used to measure color from 159 herbaceous peony cultivars	In total, 4,383,645 SLAF tags were developed from 99 P*. lactiflora* accessions, 40 SNPs were identified and significantly positively associated with petal color.	Liu et al., 2022

### Functional genes regulating flower color

**Figure 4:**
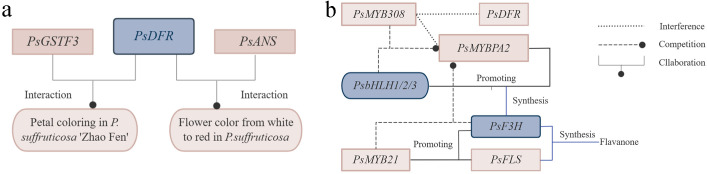
Genetic regulation of petal coloring, flower color and petal spot formation in tree peony. **(A)** Genetic regulation of petal coloring and flower color formation. **(B)** Genetic regulation of petal spot formation.

Research indicated that glutathione S-transferases transporter of anthocyanin, *PsGSTF3*, interacted with *PsDFR*, jointly promoting petal coloring in *P. suffruticosa* ‘Zhao Fen’ (Han et al., 2022). *PsDFR* and *PsANS* played crucial roles in the synthesis of flower pigments in *P. suffruticosa*, leading to the transformation of flower color from white to red (**Figure 4A**) (Zhao et al., 2015). *PsMYB58* interacted with *PsbHLH1* and *PsbHLH3* in *P. suffruticosa* ‘Er qiao’. When expressed heterologously in tobacco, *PsMYB58* promoted the expression of anthocyanin biosynthetic genes and upregulates the expression of the anthocyanin regulatory *bHLH*, *NtAN1b*, indicating that *PsMYB58* functioned as a regulator of anthocyanin biosynthesis in tree peony. *PsMYB58* played a significant role in the formation of tree peony flower color (Zhang et al., 2021a). *PsMYB44* negatively regulated anthocyanin biosynthesis by directly binding to the *PsDFR* promoter and inhibiting petal spot formation in *P. suffruticosa* ‘High noon’, thus elucidating the molecular regulatory network mediated by anthocyanins in plant spot formation (Luan et al., 2023). In the petals of *P. suffruticosa*, *PsMYBPA2* and *PsbHLH1-3* activated the expression of *PsF3H*, providing precursor substrates for anthocyanin biosynthesis and blotch formation. Meanwhile, *PsMYB21* activated the expression of both *PsF3H* and *PsFLS*, promoting flavonol biosynthesis. Its significantly high expression in the non-blotch region inhibited blotch formation by competing for substrates involved in anthocyanin biosynthesis. Conversely, its lower expression in the blotch region facilitated blotch development. Additionally, *PsMYB308* interferes with the expression of *PsDFR* and *PsMYBPA2* by competitively binding to *PsbHLH1-3* with *PsMYBPA2*, thereby preventing anthocyanin biosynthesis and blotch formation in *P. suffruticosa* petals (**Figure 4B**) (Luan et al., 2024). Exogenous glucose improved the coloration of cut flowers of *P. suffruticosa* ‘Tai Yang’ by affecting the accumulation of flavonoids and anthocyanins through signal transduction pathways. Studies indicated that ectopic expression of *PsMYB2* in tobacco led to strong pigmentation in petals and stamens, while in Arabidopsis, ectopic expression enhances the ability to accumulate anthocyanins induced by glucose in seedlings (Zhang et al., 2022b). *anthocyanin O-methyltransferase* (*PsAOMT*) and *PtAOMT*, two homologous genes, exhibited similar substrate preferences for anthocyanins *in vitro*. Transgenic tobacco expressing *PsAOMT* showed significant differences in flower color compared to wild-type tobacco. *PsAOMT* played a crucial role in flower color formation in *P. suffruticosa* ‘Gunpohden’ (purple-flowered) (Du et al., 2015).

In *P. lactiflora* ‘Hong Yan Zheng Hui’ (purplish-red), ‘Huang Jin Lun’ (yellow), and ‘Yu Lou Hong Xing’ (white), research divided the flower development process into flower-bud stage, initiating bloom, bloom stage, and withering stage. *Phenylalanine ammonia-lyase* (*PlPAL*) showed high expression levels in the flower-bud stage and withering stage. *PlCHS* and *PlCHI* maintain high expression levels throughout all developmental stages. *PlF_3_H*, *PlF_3_’H*, *PlDFR*, *PlANS*, and *UDP-glucose: flavonoid 5-o-glucosyltransferase* (*PlUF_5_GT*) gradually decreased as the flowers develop. *PlUF_5_GT* exhibited a gradual increase during flower development. Among them, *PlCHI* promoted the accumulation of chalcones, playing a crucial role in the formation of yellow flowers. The significant expression of upstream genes and low expression of *DFR* led to the synthesis of abundant anthoxanthins and a small amount of colorless anthocyanins. Due to high expressions of *PlDFR*, *PlANS*, and *PlUF_3_GT*, numerous colored anthocyanins were converted from anthoxanthins (Zhao et al., 2012a). The reduced expression of *PlF_3_′H*, *PlDFR*, and *PlANS* following daminozide treatment might have decreased anthocyanin accumulation, ultimately resulting in reduced red color intensity in *P. lactiflora* ‘Fen Zhu Pan’ (Tang et al., 2018b). Research showed that *ATP-citrate lyase* (*ACL*) *PlACLB2* promoted anthocyanin accumulation by increasing the abundance of its precursor substrate acetyl-CoA, thereby regulating the formation of red petals in *P. lactiflora* (Luan et al., 2022). Melatonin played a crucial role in the formation of herbaceous peony flower color. The tryptophan decarboxylase gene (*TDC*) gene was the rate-limiting gene in melatonin biosynthesis. Herbaceous peony petals were rich in melatonin, with the highest content found in white cultivars, followed by black, red, and pink cultivars. Melatonin levels peak during flowering, and the expression pattern of the *TDC* correlated positively with melatonin production (Zhao et al., 2018a). These studies can provided important theoretical foundations for understanding the molecular mechanisms regulating *Paeonia* flower coloration, thereby offering valuable insights for flower color improvement and breeding. Future research could further explore the differences in flower color formation among different cultivars, uncover additional key genes and transcription factors, and elucidate their regulatory networks.

## Flowering

### Flower bud dormancy

Dormancy is the primary obstacle to flowering in *Paeonia*, and studying the dormancy mechanisms of tree peonies and herbaceous peonies is of great significance for understanding their flowering mechanisms. In tree peonies, Huang et al. (2008a) screened for genes that were highly expressed in dormancy-released buds, ultimately identifying 31 unique genes, of which 25 sequences were identified as potentially involved in the dormancy release process. DNA methylation was identified as an important epigenetic regulatory factor influencing gene expression. Chilling accumulation promoted bud dormancy release and sprouting through DNA methylation modification of specific genes (Zhang et al., 2021b). Studies indicated that the *SUPPRESSOR OF OVEREXPRESSION OF CO1* (*PsSOC1*) exhibits high expression in early flowering leaves of tree peonies and showed a high hybridization signal in tender leaves of dormant buds. *PsSOC1* accelerates bud dormancy release and flowering, and its overexpression in Arabidopsis resulted in early flowering (Zhang et al., 2015b). Long-term cold exposure (0-4°C) activated the Pentose Phosphate Pathway (PPP), promoting dormancy release in *P. suffruticosa* ‘Lu he hong’ (Zhang et al., 2018b). The mitochondrial phosphate transporter gene of *P. suffruticosa* ‘Lu He Hong’, *mitochondrial phosphate transporters* (*PsMPT*), when ectopically expressed in Arabidopsis, accelerated growth and advanced flowering time. *PsMPT* played a crucial role in the dormancy release process of peony buds (Huang et al., 2008b). The transcription factor MYB-like protein, *PsMYB1*, was expressed in all tissues of *P. suffruticosa* ‘Lu He Hong’ and responds to 4°C cold temperatures. *PsMYB1* was implicated not only in dormancy release and eco-dormancy but also in tissue development (Zhang et al., 2015c). In *P. suffruticosa* ‘Lu He Hong’, *PsmiR172b* negatively regulated bud dormancy release, whereas *TARGET OF EAT* (*PsTOE3*) promoted bud dormancy release. Genes associated with bud dormancy release, including *EARLY BUD-BREAK 1* (*PsEBB1*), *EARLY BUD-BREAK 3* (*PsEBB3*), *D-type cyclins* (*PsCYCD*) and *β-1,3-glucanase* (*PsBG6*), were upregulated. Ectopic expression of *PsmiR172b-PsTOE3* in Arabidopsis conservatively regulated flowering (Zhang et al., 2023).

In herbaceous peony, the molecular mechanisms underlying dormancy release in herbaceous peony buds were studied later compared to tree peony. Zhang et al. (2015d) pioneered the construction of transcriptome libraries using RNA-seq technology for different stages of underground bud dormancy in the low-chill *P. lactiflora* ‘Hang Bai Shao’, providing a comprehensive insight into the transcriptome profiles during dormancy release. A set of target genes related to bud dormancy release were identified, involving genes associated with environmental response, energy and substance metabolism, and cell growth and development. Subsequently, analysis focused on 66 candidate genes related to light and temperature during herbaceous peony bud dormancy release, with expression pattern analysis of 12 genes across different dormancy stages, suggesting the involvement of *SOC1* and *WRKY33* in the low-temperature dormancy release process of *P. lactiflora* ‘Hang Bai Shao’ buds (Zhang et al., 2017). Subsequently, researchers utilized *P. lactiflora* ‘Da Fu Gui’ to conduct transcriptome sequencing of underground buds at three different stages: physiological dormancy, ecological dormancy, and sprouting. They identified a set of differentially expressed genes related to dormancy and focused particularly on annotating and analyzing genes associated with hormones (Guo et al., 2017).

### Flowering time

Treatment of *P. suffruticosa* ‘Qiu Fa No. 1’ with gibberellin (GA) plus nutrient can advance its autumn reflowering time by 38 days. During this process, GA induced peony reflowering, while the fertilizer provided sustained nutrients for the flowering process. At the transcriptional level, *SUPPRESSOR OF CONSTANS OF OVEREXPRESSION1* (*PsSOC1*) and *LEAFY* (*PsLFY*) played critical integrating roles in gibberellin-induced bud differentiation and nutrient supply from fertilization, respectively. *GA20 OXIDASE* (*PsGA20ox*) was primarily involved in the GA signaling pathway, while *GA INSENSITIVE* (*PsGAI*) potentially modulated flower formation post-fertilization. Sugar signaling pathways also participated in this process, emphasizing the requirement for adequate nutrient supply during early gibberellin-induced autumn reflowering of tree peonies (Xue et al., 2022). *SHORT VEGETATIVE PHASE* (*PsSVP*) negatively regulated flowering and positively regulated leaf growth in *P. suffruticosa* ‘Luoyang Hong’. *Gibberellic ACID 3* (GA_3_) improved floral bud development by suppressing *PsSVP* expression. High expression of the *PsSVP* gene may contributed to floral bud abortion in tree peonies (Wang et al., 2014).

The leaves of *P. ostii* ‘Fengdan’ were sprayed with brassinolide at concentrations of 25 μg/L, 50 μg/L, 100 μg/L, and 200 μg/L. Significant flowering delay was observed with brassinolide at 50 μg/L. Transcriptome and miRNA sequencing of *P. ostii* ‘Fengdan’ leaves treated with different brassinolide concentrations identified six miRNAs and their target genes potentially involved in regulating the flowering of *P. ostii*. These include four novel miRNAs targeting *SPA1*, miR156b targeting *SPL*, and miR172 targeting *AP2* (Zhang et al., 2022c). Additionally, research indicated that in *P. ostii* ‘Fengdan’, transgenic plants with *pCAMBIA2300-PoVIN3* exhibit significantly earlier flowering compared to the wild type, suggesting a role for the *PoVIN3* gene in promoting flowering (Li et al., 2022b). Overexpression of the *PoFLC* gene in Arabidopsis resulted in significantly delayed bolting and flowering compared to the wild type, indicating an important role for the *PoFLC* gene in delaying flowering processes (Fan et al., 2023b). Overexpression of the gene *CONSTANS* (*PlCO*) from *P. × lemoinei* ‘High Noon’ in *Arabidopsis* resulted in earlier flowering, indicating its promotive role in tree peony flowering. PlCO protein localized to the nucleus and exhibited transcriptional activity, suggesting that PlCO may function as a transcription factor (Chang et al., 2022). Transcriptome sequencing was conducted on *P. lemoinei* ‘High Noon’ and non-reblooming *P. suffruticosa* ‘Luo Yang Hong’, identifying 59,275 and 63,962 unigenes, respectively. Integrated analysis and RT-PCR validation identified several genes potentially playing crucial roles in tree peony reblooming, including *FLOWERING LOCUS T* (*PsFT*), *VERNALIZATION INSENSITIVE 3* (*PsVIN3*), *PsCO* and *GIBBERELLIN 20-OXIDASE* (*PsGA20OX*) (Zhou et al., 2013).

Dormancy breaking treatment of *P. lactiflora* ‘Taebaek’ in November (0°C for 6 weeks) advanced flowering to early spring. However, similar treatments in August, September, or October resulted in flower bud abortion or deformities. Research indicated that a 2-week pre-chilling at 10°C followed by dormancy breaking treatment was the optimal method for promoting autumn or winter flowering of herbaceous peonies without bud abortion (Park et al., 2015).

### Bloom period of cut flowers

Research indicated that the duration of cut flower bloom period was closely related not only to harvest time and storage methods but also to the addition of exogenous substances post-harvest, which could enhance cut flower quality and prolong the bloom period. During the cut flower opening process of *P. suffruticosa* ‘Luoyang Hong’, studies have found that ethylene inhibits the expression of *Ethylene resistant* (*Ps-ETR1-1*) and *Ethylene insensitive* (*Ps-EIN3-1*) genes. Additionally, *Ps-ETR1-1* expression was significantly inhibited by 1-MCP, with a slight induction observed in *Ps-EIN3-1* gene expression. This suggests that the proteins encoded by *Ps-ETR1-1* and *Ps-EIN3-1* played important roles in ethylene sensitivity and response processes in tree peony cut flowers. These findings were crucial for extending the cut flower bloom period (Zhou et al., 2010). Furthermore, research indicated that *PsEIL2*, *PsEIL3*, and *PsERF1* might also have played roles in regulating ethylene response in *P. suffruticosa* ‘Luoyang Hong’ (Wu et al., 2017).

Treatment of fresh-cut flowers of *P. lactiflora* ‘Hongyan Zhenghui’ with nano-silver increased the soluble protein content inside the flowers. This enhancement promoted the activities of antioxidant enzymes such as superoxide dismutase (SOD), catalase (CAT), and ascorbic acid peroxidase (APX), thereby reducing the accumulation of reactive oxygen species such as malondialdehyde (MDA), superoxide radicals (O_2_^·−^), and hydrogen peroxide (H_2_O_2_). Furthermore, nano-silver treatment exerted inhibitory effects on the growth and reproduction of microorganisms at the stem base of cut flowers, thereby delaying stem blockage. Additionally, *AQUAPORIN* genes (*AQPs*) induced by nano-silver treatment played a crucial role in maintaining water balance in cut flowers (Zhao et al., 2018b). Moreover, research has shown that both sucrose transporter genes and invertase genes were involved in maintaining vase quality in cut flowers, specifically in *P. lactiflora* ‘Yang Fei Chu Yu’ (Xue et al., 2018).

Silencing *PlZFP* in tobacco petals prolonged flower opening time, while overexpressing *PlZFP* shortened flower lifespan. This process involved downregulation of genes related to plant hormone biosynthesis, including *PlACO1*, *PlACO3*, *PlACS1*, *PlNCED2* and *PlAAO*, along with increased transcript levels of *PlGA3ox1*. Conversely, overexpression of *PlZFP* in tobacco flowers exhibited opposite effects on these genes. Additionally, *PlZFP* directly bound to the *PlNCED2* promoter. Silencing *PlNCED2* delayed senescence in petal discs. Reduction of *PlZFP* decreased ethylene and ABA levels but increased GA levels in herbaceous peony petal discs. Furthermore, exogenous ABA, ethephon, and paclobutrazol (PAC), a GA inhibitor, accelerated senescence in *PlZFP*-silenced discs. Collectively, the *PlZFP-PlNCED2* regulatory checkpoint likely enhanced ABA production and modulates the interplay of ABA with GA and ethylene during flower senescence in cut *P. lactiflora* ‘Hangshao’ (Ji et al., 2023).

## Floral fragrance

### Preparation, analysis, and components of floral fragrance

*Paeonia* are known for their rich floral fragrances, with various aromatic profiles identified in tree peony flowers, including grassy scent, fruity scent, woody scent, rose scent, lily of the valley scent, phenolic scent, and unidentified scent (Li et al., 2012, 2021). In herbaceous peony fragrance research, six types of fragrances have been identified, namely woody scent, fruity scent, lily scent, rose scent, orange blossom scent, and mixed scent (Song et al., 2018; Zhao et al., 2023).

The composition of floral fragrance was complex and diverse, primarily including alcohols, aldehydes, terpenes, esters, and volatile phenols, which often exhibit significant diversity in plants (Li et al., 2012; Luo et al., 2020). Currently, common methods for aroma collection and component analysis in *Paeonia* included Headspace Solid-Phase Microextraction (HS-SPME) and Dynamic Headspace Sampling (DHS). Widely used analytical methods included artificial olfactory systems, GC-MS, and Automated Thermal Desorber-Gas Chromatography-Mass Spectrometry (ATD-GC/MS) (**Table 5**) (Zhao et al., 2012b; Li et al., 2023). The headspace technique was favored in fragrance preparation due to its automation, ability to minimize sample matrix interference, and simplicity in sampling, thus it was widely applied in volatile compound preparation. GC-MS offered high sensitivity and simultaneous qualitative and quantitative analysis capabilities, contributing to its widespread use in fragrance component analysis.

**Table 5:** Preparation, analysis, and composition of fragrance in *Paeonia* flowers.

Plant materials	Scent collection	Scent analysis	Components of floral fragrance	References
6 tree peony cultivars	DHS	ATD-GC/MS	105 types of volatile compounds from 10 classes were identified, with the most abundant volatiles being alpha-pinene, 2,3-dihydroxy propanal, 3-methyl-1-butanol, 2-ethyl-1-hexanol, acetic acid 1-methylethyl ester, and 5-ethyl-2,2,3-trimethyl heptane.	Zhao et al., 2012b
97 tree peony cultivars with various genetic backgrounds	HS-SPME	GC-MS	56 volatile compounds were detected, including 26 terpenoids, 20 fatty acid derivatives, nine benzenoids/phenylpropanoids, and one amino acid derivative.	Li et al., 2023
36 *P. suffruticosa* cultivars from four cultivar-groups	DHS	ATD-GC/MS	128 volatile components were identified from the petals, and it was found that they consisted primarily of terpenes, alcohols, and esters.	Li et al., 2021
30 Paeonia × suffruticosa cultivars from six cultivar-groups	HS-SPME	GC-MS	146 volatile organic components were identified, with 81 compounds confirmed as scent components in 30 tree peony cultivars. The major scent components included terpenes, alcohols, arenes, and alkanes.	Li et al., 2012
87 herbaceous peony (Paeonia lactiflora Pall.) cultivars from three cultivar-groups	HS-SPME	GC-MS	26 major aroma compounds were identified, including terpenoids, benzenoids/phenylpropanoids, and fatty acid derivatives. Linalool, geraniol, citronellol, and phenylethyl alcohol (2-PE) were identified as characteristic substances contributing to the aroma of herbaceous peonies.	Zhao et al., 2023
30 herbaceous peony cultivars from four groups	DHS	ATD-GC/MS	130 volatile compounds were identified, with 72 of them being aroma compounds. The main compounds include phenylethyl alcohol, β-caryophyllene, linalool, (R)-citronellol, and nerol.	Song et al., 2018
9 wild tree peony species	HS-SPME	GC-MS	124 volatile compounds belonging to five categories: terpenoids, alkanes, alcohols, aldehydes, and ketones, were identified.	Luo et al., 2020
*P. lactiflora* ‘Wu Hua Long Yu’ at different stages of flower development	HS-SPME	GC-MS	Key regulators of monoterpene synthesis in herbaceous peony appear to be 1-deoxy-D-xylulose 5-phosphate synthase (DXS), geranyl pyrophosphate synthase (GPPS), and terpene synthase (TPS).	Zhao et al., 2024

The fragrance composition of *Paeonia* was complex and diverse (**Table 5**). In tree peonies, the main components responsible for the grassy scent in petals were ocimene, for fruity scent it was 2-ethyl hexanol, for musky scent it was cis-ocimene/longifolene, for rose scent it was D-citronellol, for lily scent it was linalool, for phenolic scent it was 1,3,5-trimethoxybenzene, and for unknown scent it was Pentadecane (Li et al., 2012, 2021). In herbaceous peonies, the primary components responsible for woody scent were β-caryophyllene, for fruity scent it was phenylethyl alcohol, for lily scent it was linalool, for rose scent it was (R)-citronellol, and for orange blossom scent it was nerol (Song et al., 2018; Zhao et al., 2023). Additionally, terpenes were the most abundant compounds in peony petal volatiles (Li et al., 2012), and major fragrance components in peonies also include geraniol and 2-phenylethyl alcohol (Zhao et al., 2023). In conclusion, the fragrance components of *Paeonia* are complex and diverse, and further research is needed to understand their biosynthetic pathways and gene regulation mechanisms.

### Flower fragrance molecular biology study

*Paeonia* flower fragrance is a quantitatively controlled trait influenced by multiple genes, with a complex genetic regulatory mechanism. Studying the biosynthetic pathways of floral fragrance compounds is crucial for understanding the molecular mechanisms underlying fragrance formation in peonies. In tree peonies, linalool was identified as one of the dominant volatile compounds in the fragrance of members of the subsection Delavayanae and subsect. Delavayanae hybrids. Five terpene synthases (TPSs) (*PdTPS1*, *PdTPS2*, *PdTPS3*, *PdTPS4*, and *PdTPS5*) were identified through global transcriptome sequencing analysis of 11 wild tree peony genotypes and 60 cultivars, potentially involved in the biosynthetic pathway of linalool. Among these, *PdTPS1*, *PdTPS2*, and *PdTPS4* showed significant positive correlations with linalool emissions across tree peony cultivars. Biochemical data analyses confirmed that *PdTPS1* and *PdTPS4* were key genes determining linalool synthesis (Li et al., 2023). Furthermore, expression levels of enzymes crucial for terpene skeleton biosynthesis, including acetoacetyl-CoA thiolase (AACT), 3-Hydroxy-3-methylglutaryl-coenzyme A reductase (HMGR), phosphomevalonate kinase (PMK), 1-Deoxyxylulose-5-phosphate synthase (DXS), 1-deoxy-D-xylulose-5-phosphate reductoisomerase (DXR), 4-Hydroxy-3-methylbut-2-enyl diphosphate synthase (HDS), 4-hydroxy-3-methylbut-2-enyl diphosphate reductase (HDR), and geranylgeranyl diphosphate synthase (GGPS), were upregulated in the tree peony cultivar ‘Huangguan’ (strong fragrance) compared to the cultivar ‘Fengdan’ (faint fragrance). Additionally, transcription abundance of one linalool synthase gene (LIS) and one myrcene synthase gene (MYS) in ‘Huangguan’ petals was higher compared to ‘Fengdan’. Therefore, high expression of genes involved in terpene skeleton biosynthesis and monoterpene metabolism pathways was associated enhanced fragrance in tree peonies (Li et al., 2021).

In herbaceous peonies, analysis of aroma components and aroma thresholds across 17 cultivars identified linalool, geraniol, citronellol, and phenylethyl alcohol (2-PE) as primary aromatic substances. Subsequent qRT-PCR investigations into genes potentially regulating aroma synthesis in peony petals revealed significant candidates, including 1-DEOXY-D-XYLULOSE 5-PHOSPHATE SYNTHASE (*PlDXS2*), 1-deoxy-D-xylulose-5-phosphate reductoisomerase (*PlDXR1*), 2-C-METHYL-D-ERYTHRITOL-2,4-CYCLODIPHOSPHATE (*PlMDS1*), 1-HYDROXY-2-METHYL-2-(E)-BUTENYL-4-DIPHOSPHATE REDUCTASE (*PlHDR1*), GERANYL PYROPHOSPHATE SYNTHASE (*PlGPPS3* and *PlGPPS4*), as well as LINALOOL SYNTHASE (*LIS*) and GERANIOL SYNTHASE (*GES*) genes. These findings underscored the critical role of differential gene expression in the monoterpene and 2-PE synthesis pathways in shaping the aroma profile of herbaceous peonies (Zhao et al., 2023).

Furthermore, improvement of floral fragrance traits in *Paeonia* requires support from genomic marker resources. Molecular marker studies on floral fragrance traits in *Paeonia* have been shown to promote the breeding of fragrant cultivars. Research based on tree peony genomic data has developed microsatellite markers associated with major floral fragrance components. A total of 8443 EST-SSRs were identified, with genetic diversity and population structure analyses conducted on 31 polymorphic EST-SSR markers, yielding 176 alleles. Polymorphic information content (PIC) ranged from 0.31 to 0.87. Single marker-trait association analysis using a mixed linear model (MLM) identified 29 significantly associated loci, involving 18 SSR loci associated with 5 floral fragrance components, with phenotypic variance explained ranging from 4.57% to 23.46% (Luo et al., 2021b).

## Discussion

While numerous scholars have identified genes potentially involved in *Paeonia* floral development, how these genes function remains unclear, and the expression patterns of these genes and their interactions have not been thoroughly investigated. The mechanisms and formation processes of floral development in *Paeonia* are not well understood. Future research should focus on elucidating the interactions between the genes that regulate floral development in peonies.

The formation of flower color in *Paeonia* is a complex process involving interactions among multiple genes and regulatory networks. Despite the identification of key genes and regulatory factors, the genetic regulatory network underlying flower color formation remains incompletely understood. Further research is needed to investigate the interactions among different genes and their regulatory mechanisms. Current studies have primarily focused on a few *Paeonia* cultivars, necessitating deeper exploration into the differences in flower color formation among different cultivars and the corresponding genetic variations. Future research should delve into the interactions among different genes to construct a comprehensive regulatory network for peony flower color formation. This will uncover more critical regulatory factors and pathways. Additionally, studying the impact mechanisms of environmental factors such as light and temperature on *Paeonia* flower color formation will provide more possibilities and approaches for regulating *Paeonia* flower color.

In the study of flowering in *Paeonia*, there is a lack of comprehensive understanding regarding the release of flower bud dormancy and flowering, and a need for deeper exploration into the functions of key genes and regulatory factors. The molecular mechanisms underlying physiological and ecological dormancy are also poorly understood. Furthermore, research on the influence of environmental factors such as temperature and light on the flowering of *Paeonia* is relatively scarce, and their regulatory mechanisms remain incompletely elucidated. Despite some understanding of cut flower quality, ethylene responses, and their regulatory mechanisms in *Paeonia*, there are still deficiencies. Future research efforts should focus on elucidating the molecular mechanisms governing the release of flower bud dormancy, exploring novel regulatory factors and gene families, and further clarifying the regulatory networks controlling the flowering in *Paeonia*. Additionally, there should be enhanced studies on the differences in flowering among different cultivars, and a deeper investigation into the effects of environmental factors on flowering to enhance understanding and optimize cultivation techniques in *Paeonia*. In terms of fresh cut flower preservation, there is a need for in-depth research into the cut flower characteristics of different peony species and their ethylene signaling pathways, to broaden our understanding of herbaceous peony cut flowers. Furthermore, research should investigate the mechanisms by which exogenous substances affect the quality of herbaceous peony cut flowers during storage, aiming to extend the storage period and prolong the bloom period.

Research on the complexity of fragrance components and the related biosynthetic pathways in the study of floral fragrance in *Paeonia* remains unclear. Additionally, further exploration is needed into the genetic regulatory networks governing floral fragrance traits, and the development and utilization of genomic marker resources for fragrance-related genes in *Paeonia* have not been fully realized. Future research can delve deeply into identifying fragrance-related genes in *Paeonia*, exploring details of fragrance biosynthetic pathways, enhancing genetic resource studies among cultivars, and employing advanced molecular biology and gene editing technologies for precise breeding to improve fragrance quality and yield in *Paeonia*.

Research has found that in terms of flower shape and petal quantity, tree peonies are more complex and abundant, while herbaceous peonies are relatively simpler. In terms of color diversity and vibrancy, tree peonies are generally more prominent. Tree peonies bloom earlier, while herbaceous peonies bloom slightly later. There are also differences in fragrance strength and type, with tree peonies typically having a more intense scent. Peonies exhibit a variety of floral scents and rich colors, as well as differences in blooming periods and fragrances. Comparing floral traits qualitatively or quantitatively among different species or varieties can be challenging. Future research could delve deeper into the mechanisms behind the differences in flower shape, color, blooming period, and fragrance among various species or varieties.

In summary, many scholars have identified key genes regulating flower shape, color, blooming, and fragrance in the Peony genus. However, the specific functions of these genes and their interactions require further investigation. Additionally, the key pathways and gene regulatory networks controlling flower development are not yet clear, and future research should focus on these areas. With the maturity of CRISPR/Cas9 technology, its application in plant research is becoming increasingly widespread, including flower development and plant growth and development (Fan et al., 2020). In the future, using CRISPR/Cas9 technology to artificially modify the floral organ traits of *Paeonia* is of great significance for screening and cultivating ornamental plants with high economic value, as well as for expanding the cultivation range and introducing new cultivars.
